# Preparations of NiFe_2_O_4_ Yolk-Shell@C Nanospheres and Their Performances as Anode Materials for Lithium-Ion Batteries

**DOI:** 10.3390/nano10101994

**Published:** 2020-10-09

**Authors:** Tianli Liu, Qinghua Gong, Pei Cao, Xuefeng Sun, Jing Ren, Shaonan Gu, Guowei Zhou

**Affiliations:** Key Laboratory of Fine Chemicals in Universities of Shandong, School of Chemistry and Chemical Engineering, Qilu University of Technology (Shandong Academy of Sciences), Jinan 250353, China; 17862979520@163.com (T.L.); 18396814931@163.com (Q.G.); caopei8956@126.com (P.C.); sunxf0210@163.com (X.S.); 17862963042@163.com (J.R.)

**Keywords:** NiFe_2_O_4_, yolk-shell structure, carbon coating, lithium-ion batteries

## Abstract

At present, lithium-ion batteries (LIBs) have received widespread attention as substantial energy storage devices; thus, their electrochemical performances must be continuously researched and improved. In this paper, we demonstrate a simple self-template solvothermal method combined with annealing for the synthesis of NiFe_2_O_4_ yolk-shell (NFO-YS) and NiFe_2_O_4_ solid (NFO-S) nanospheres by controlling the heating rate and coating them with a carbon layer on the surface via high-temperature carbonization of resorcinol and formaldehyde resin. Among them, NFO-YS@C has an obvious yolk-shell structure, with a core-shell spacing of about 60 nm, and the thicknesses of the NiFe_2_O_4_ shell and carbon shell are approximately 15 and 30 nm, respectively. The yolk-shell structure can alleviate volume changes and shorten the ion/electron diffusion path, while the carbon shell can improve conductivity. Therefore, NFO-YS@C nanospheres as the anode materials of LIBs show a high initial capacity of 1087.1 mA h g^−1^ at 100 mA g^−1^, and the capacity of NFO-YS@C nanospheres impressively remains at 1023.5 mA h g^−1^ after 200 cycles at 200 mA g^−1^. The electrochemical performance of NFO-YS@C is significantly beyond NFO-S@C, which proves that the carbon coating and yolk-shell structure have good stability and excellent electron transport ability.

## 1. Introduction

Lithium-ion batteries (LIB) have attracted tremendous attention as practical energy storage devices in recent decades [1]. As an indispensable component of a battery, the anode is critical to the performance of LIB. Recently, more attempts have been made in materials to explore high-performance anodes for LIB beyond traditional graphite and analogues, including carbon materials [2,3], transition metal oxides [4,5], silicon materials [6,7], and even alloy materials [8]. In particular, spinel structured oxides (AB_2_O_4_) exhibit superior electrochemical performance compared with the corresponding single-component oxides due to the synergy between different metal ions, unique crystal structure, multiple oxidation states, and other characteristics [9,10,11,12]. However, spinel AB_2_O_4_ also has shortcomings such as poor conductivity and volume expansion during charging and discharging, which lead to the problems of large irreversible capacity and poor cycle performance in LIB [13,14]. Therefore, there is still a long way to go for the exploration of AB_2_O_4_ anode materials.

People have adopted various strategies to improve the electrochemical performance of AB_2_O_4_ materials. For instance, metal and non-metal doping or substitution can improve thermal stability and reaction kinetics [15,16,17], and the introduction of heterojunction improves Li^+^ diffusion rate and electron conduction rate [18,19]. In addition, coupling with highly conductive carbon-based materials can effectively improve the conductivity of AB_2_O_4_, such as graphene [20,21,22,23], carbon nanotubes [24,25,26,27], and others [28,29]. Another feasible solution is carbon coating on the surface of AB_2_O_4_, which improves the electronic conductivity of the material and also generates a shielding layer to block the internal substance from directly contacting with electrolyte, thereby inhibiting the continuous reconstruction of the solid electrolyte interface (SEI) film [30,31]. Therefore, carbon coating can effectively improve the conductivity and cycle performance of AB_2_O_4_ materials.

Additionally, designing the structure of AB_2_O_4_ is also one of the most useful methods for improving the electrochemical performance. The hollow structure has the advantages of low density, large surface area, and reduced charge transfer length, which is been proven to have huge structural potential in energy storage and conversion [32,33,34]. Recently, hollow NiCo_2_O_4_ nanospheres [35], hollow ZnFe_2_O_4_ nanospheres [36], hollow ZnCo_2_O_4_ octahedrons [37], and hollow NiCo_2_O_4_ polyhedrons [38] have been synthesized for LIB anode materials. The reversible capacities of the hollow structure anode matrix were more raised up than that of the solid structure. However, a reasonable increase in the complexity of the hollow structure may bring better electrochemical performance than the simple hollow structure [39]. The yolk-shell structure could be a promising candidate as it not only possesses the advantages of the hollow structure such as short ion/electron transmission distance and unique surface and pore structure, but it also has a high active material weight fraction and provides durable transfer channels for ions and electrons. Assuming that the AB_2_O_4_ yolk-shell structure is designed and combined with the modest surface carbon coating, the electrochemical performance of the AB_2_O_4_ material could be improved reasonably.

Herein, this work designed and synthesized carbon-coated NiFe_2_O_4_ (NFO) yolk-shell nanospheres (NFO-YS@C) for LIB anode materials. NiFe_2_O_4_ has a unique inverse spinel structure. Ni^2+^ and one-half of Fe^3+^ are distributed in octahedral voids, and the remaining of Fe^3+^ occupies tetrahedral positions [40]. This structure has a high theoretical capacity because NFO can hold eight Li^+^ per unit during the lithiation/delithiation process [41,42]. The gap between the tetrahedron and the octahedron can be used as a three-dimensional ion transport channel, which is conducive to electrochemical reactions. For the results, NFO-YS@C has a relatively high capacity of 1023.5 mA h g^−1^ at 200 mA g^−1^ after 200 cycles and good cycling performance, and the electrode could also deliver 400.4 mA h g^−1^ capacity even at the current density of 1000 mA g^−1^, which was assigned to the combination of yolk-shell structure and surface carbon coating.

## 2. Materials and Methods

### 2.1. Materials and Chemicals

Nickel(Ⅱ) nitrate hexahydrate (Ni(NO_3_)_2_ × 6H_2_O), Iron(Ⅲ) nitrate nonahydrate (Fe(NO_3_)_3_ × 9H_2_O) and resorcinol were purchased from Sigma-Aldrich (St. Louis, MO, USA). Isopropyl alcohol, glycerol, formaldehyde (37 wt.%), ethanol and ammonium hydroxide (NH_3_ × H_2_O, 28 wt.%) were purchased from Sinopharm Chemical Reagent Co., Ltd. (Shanghai, China). 

### 2.2. Synthesis of NiFe_2_O_4_ Yolk-Shell Nanospheres

First, 0.0363 g of Ni(NO_3_)_2_ × 6H_2_O and 0.101 g of Fe(NO_3_)_3_ × 9H_2_O were added to the mixed solvents glycerol (8 mL) and isopropanol (40 mL), resulting in a clear orange-yellow liquid. Then, the liquid was poured into an autoclave and heated to 180 °C for 6 h. The yellow NiFe-glyceric acid precursor was collected, washed thoroughly with ethanol, and dried in air at 60 °C. The as-synthesized NiFe-glycerate precursors were annealed at 400 °C in air for 2 h at a slow heating rate of 1 °C min^−1^ to form NFO-YS nanospheres. The NFO-S nanospheres were synthesized through the above procedure by changing the heating rate to 2 °C min^−1^.

### 2.3. Synthesis of NiFe_2_O_4_@C Nanospheres

First, 50 mg NFO-YS was added to 20 mL ethanol and 10 mL deionized water, and sonicated for 10 min. Then, under mechanical stirring, 0.5 mL of NH_3_ × H_2_O solution was added to the above solution and reacted for 10 min, and then 0.1 g of resorcinol and 0.12 mL of formaldehyde were added. After stirring at room temperature for 2 h, a layer of resorcinol and formaldehyde resin (RF) was formed on the surface of the NFO-YS. The collected NFO-YS@RF nanospheres were washed with ethanol, dried, and annealed under Ar at 600 °C for 2 h to obtain the NFO-YS@C nanospheres. NFO-S@C nanospheres were synthesized by the above procedure with NFO-S nanospheres.

### 2.4. Characterization

The morphology and structure of the sample were observed using a field emission scanning electron microscope (FESEM), transmission electron microscope (TEM), and high-resolution TEM (HRTEM). X-ray diffraction patterns (XRD) were collected on an X-ray spectrometer (RigkuSE, Tokyo, Japan). Raman spectra were measured by laser confocal Raman spectrometer (Renishaw in Via9, London, UK). X-ray photoelectron spectroscopy (XPS) data were recorded on an X-ray photoelectron spectrometer (ESCALABXi₊, Waltham, MA, USA). The N_2_ adsorption–desorption isotherms and the corresponding pore size distributions were obtained through the specific surface and porosity analyzer (Micromeritic ASAP 2400, Norcross, GA, USA). Thermogravimetric analysis (TGA) was performed via a synchronous thermal analyzer (Netzsch STA449 F3, Selb, Germany) under air flow.

### 2.5. Electrochemical Measurements

The as-prepared samples (70 wt.%), acetylene black (20 wt.%), and polyvinylidene fluoride (10 wt.%) were ground and mixed. After adding the N-methyl 2-pyrrolidine solvent, the electrode materials were ground evenly using a planetary ball mill. The uniformly mixed anode material was coated on Cu foil, and the mass load of the sample was about 1.0–1.5 mg cm^−2^. After the electrode film was completely dried, it was cut into 12 mm diameter round flakes with a microtome. The preparation of the coin cell was performed in a glovebox and argon-filled. The electrolyte was 40 µL of 1.0 mol L^−1^ LiPF_6_ in EC, DMC, and EMC (1:1:1, volume ratio). High-purity lithium foil was used as the counter electrode and Celgard 2400 (Charlotte, NC, USA) as the separator. Cyclic voltammetry (CV) and electrochemical impedance spectroscopy (EIS) were obtained on a electrochemical workstation (PARSTAT 4000, Berwyn, PA, USA). The testing of coin cells was performed on a multi-channel battery test system (LAND-CT2001A, Wuhan, China) in the voltage range of 3.00 to 0.01 V versus Li^+^/Li.

## 3. Results and Discussion

**Figure 1:**
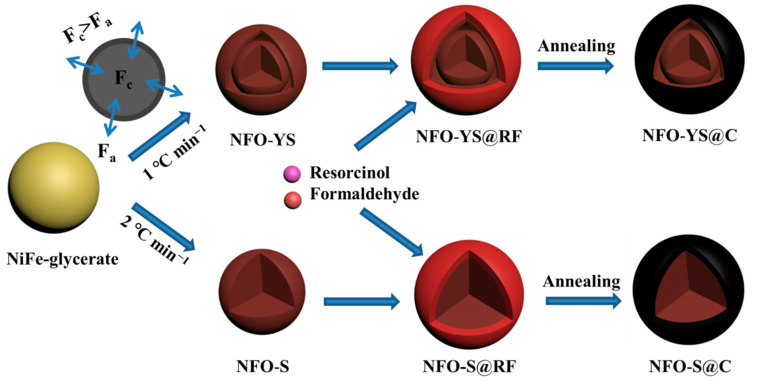
Schematic illustration of the formation process of NFO-YS@C and NFO-S@C.

Figure 1 illustrates the strategy for preparing NFO-YS@C and NFO-S@C nanospheres. First, NiFe-glycerate nanospheres were prepared using the solvothermal method [43,44]. The hydroxide ion release by the oxidation-reduction reaction of isopropanol and NO^3−^ caused Ni^2+^ and Fe^3+^ to precipitate as Ni-Fe double hydroxides, resulting in uniform NiFe-glycerate nanospheres. Next, the NiFe-glycerate nanospheres were transformed into NiFe_2_O_4_ nanospheres with different internal structures through simple non-equilibrium heat treatment. When the heating rate was 1 °C min^−1^, the formation of NiFe_2_O_4_ shell was carried out on the surface of NiFe-glycerate in the initial stage of calcination. At this time, two reaction forces were between the rigid NiFe_2_O_4_ shell and the NiFe-glycerate, and the contraction force (Fc) was induced by the oxygenolysis of organic matter and the adhesion force (Fa) of the external rigid shell [45,46]. Fc caused NiFe-glycerate to contract inward, while Fa prevented it from contracting inward. When Fc exceeded Fa, NFO-YS nanospheres in the form of yolk-shell were formed. However, when the heating rate was increased, a larger temperature gradient resulted in the NiFe_2_O_4_ shell forming faster on the surface of the NiFe-glyceride core during the initial annealing, and the inner core had no time to detach from the outer shell, thereby forming the solid spherical NFO-S. The surface was coated with a carbon layer to avoid the collapse of the structure of NiFe_2_O_4_ during charging and discharging. Resorcinol and formaldehyde underwent condensation and polycondensation reactions on the surface of NiFe_2_O_4_ nanospheres to form an RF layer. NFO-YS@C and NFO-S@C nanospheres were obtained by carbonization through high-temperature calcination.

**Figure 2:**
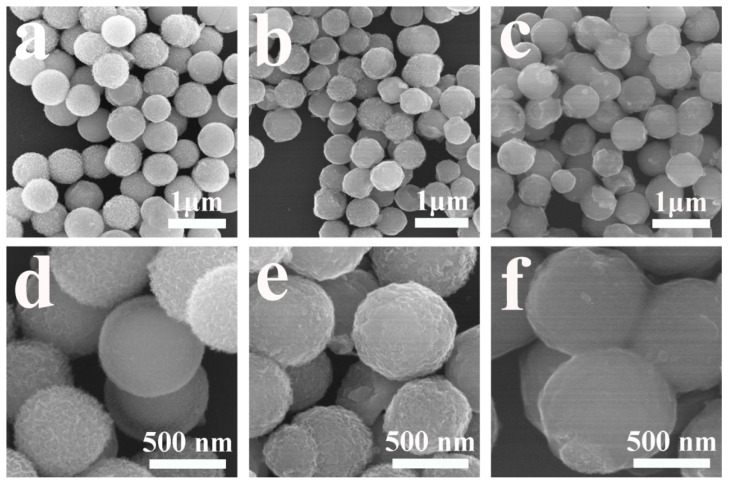
FESEM images of (**a**,**d**) NFO-YS, (**b**,**e**) NFO-S, and (**c**,**f**) NFO-YS@C.

FESEM images in Figure 2 show that the prepared samples consist of numerous uniformly sized nanospheres. Figure 2a,b are FESEM images of NFO-YS and NFO-S, both having similar morphology and the same average diameter of 650 nm. The surfaces of NFO-YS and NFO-S nanospheres are relatively rough (Figure 2d,e) because NiFe-glycerate nanospheres underwent a hydrolysis reaction to form hydroxide nanosheets in the solvent [47]. Figure 2c,f show FESEM images of NFO-YS@C nanospheres with different magnifications. Compared with NFO-YS and NFO-S, the diameters of NFO-YS@C and NFO-S@C (Figure S1) nanospheres are substantially large, about 710 nm, and the surfaces are relatively smooth, indicating that the carbon layer covers the surface of NFO-YS uniformly.

**Figure 3:**
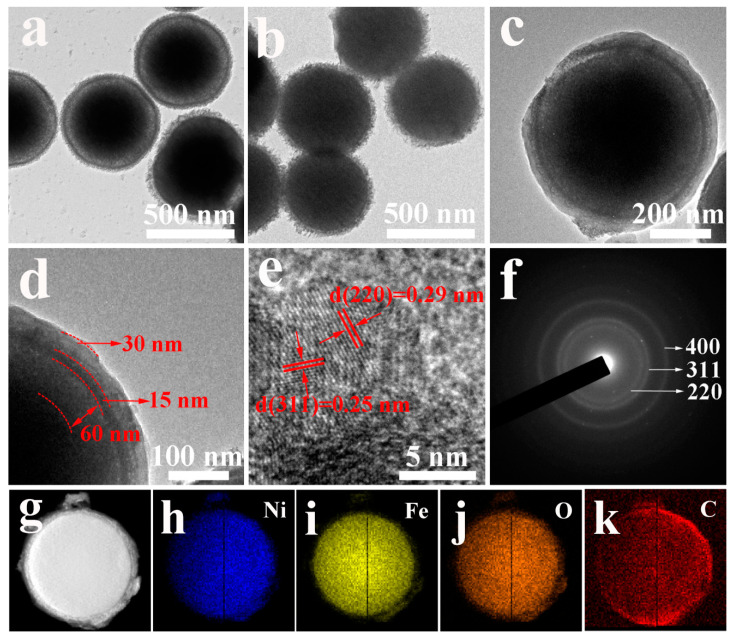
TEM images of (**a**) NFO-YS, (**b**) NFO-S, and (**c**,**d**) NFO-YS@C. HRTEM image of (**e**) NFO-YS@C. SAED pattern of (**f**) NFO-YS@C. (**g**) STEM and (**h**–**j**) elemental mapping images of Ni, Fe, O, and C in NFO-YS@C.

TEM images of Figure 3a,b show NFO-YS with a yolk-shell structure and NFO-S with a solid sphere structure are obtained by calcining NiFe-glycerate precursors (Figure S2) at different heating rates, respectively. When the heating rate changed from 1 °C min^−1^ to 2 °C min^−1^, the structure of the sample changed from a typical yolk-shell sphere structure (Figure 3a) to a solid sphere structure (Figure 3b). The TEM images of NFO-YS@C in Figure 3c and NFO-S@C in Figure S3 show that the surfaces of NFO-YS and NFO-S are covered with a uniform carbon shell. After calcination at high temperature, the inner structures of NFO-YS@C and NFO-S@C will not be destroyed. Figure 3d shows the magnification TEM image of NFO-YS@C nanospheres. The apparent inter-lamellar gap between the core and shell is approximately 60 nm, and the thicknesses of the shell and carbon layer are approximately 30 and 15 nm, respectively. Figure 3e shows the HRTEM image of an individual NFO-YS@C. The lattice spacings of 0.29 and 0.25 nm correspond to (220) and (311) d-spacing of the NiFe_2_O_4_ species. The selected area electron diffraction (SAED) mode (Figure 3f) shows three distinct rings, which are consistent with the crystal plane of NiFe_2_O_4_, indicating that the crystallinity of NFO-YS@C is good. In addition, the energy-dispersive spectrometry mapping images (Figure 3g–k) confirm the uniform distribution of Ni, Fe, and O elements in NFO-YS@C, while the C element is concentrated in the outermost layer of the nanospheres.

**Figure 4:**
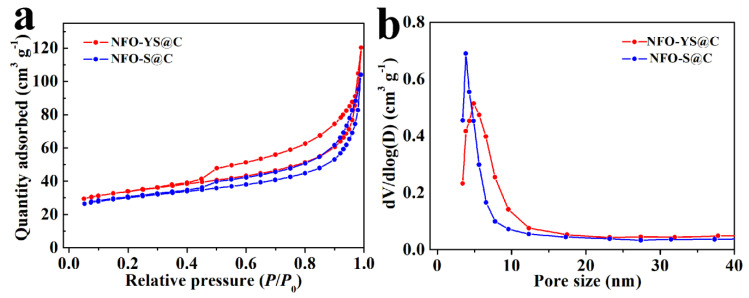
(**a**) The N_2_ adsorption–desorption isotherms of NFO-S@C and NFO-YS@C, (**b**) the corresponding pore size distributions.

Studies have shown that the existence of the porous structure can accelerate the dynamic process of ion diffusion in the structure, and a large specific surface area can increase the contact area between the electrode and the electrolyte and improve electrochemical performance [48,49]. In order to compare the specific surface area and pore size of NFO-YS@C and NFO-S@C, the N_2_ adsorption–desorption isotherms and pore size distributions are shown in Figure 4. The isotherms of NFO-YS@C and NFO-S@C exhibit type IV with H1 hysteresis behaviors caused by the characteristics of mesoporous morphology [45,50]. It can be seen from the pore size distribution in Figure 4b that the pore diameters of NFO-YS@C and NFO-S@C are mainly distributed between 4−10 nm. The specific pore diameter of NFO-YS@C is about 8.03 nm and the pore volume is 0.25 cm^3^ g^−1^, while the pore diameter of NFO-S@C is approximately 6.37 nm, the pore volume is 0.21 cm^3^ g^−1^, and the corresponding specific surface area is 141.13 and 110.87 m^2^ g^−1^, respectively. The results show that NFO-YS@C has a relatively large specific surface area and pore size, which may be attributed to the internal yolk-shell structure. The large specific surface area and pore size of NFO-YS@C provide a good structural basis for the diffusion of lithium ions and the penetration of electrolytes during charge and discharge.

**Figure 5:**
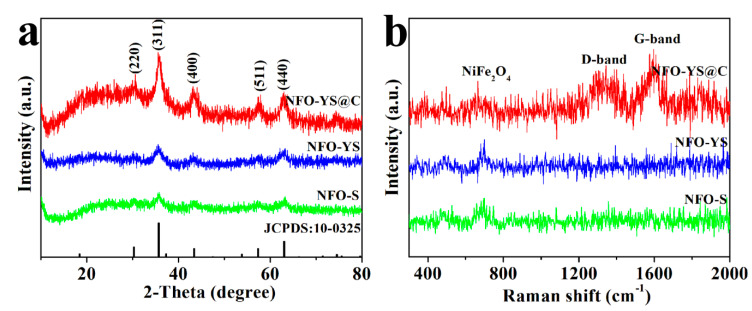
XRD patterns (**a**) and Raman spectra (**b**) of NFO-YS, NFO-S, and NFO-YS@C.

Figure 5a shows XRD patterns of the prepared NFO-YS, NFO-S, and NFO-YS@C nanospheres. For NFO-YS and NFO-S, the diffraction peaks at 30°, 36°, 43°, 57.5°, and 63° represent the (220), (311), (400), (511), and (440) crystal planes of the cubic structure of NiFe_2_O_4_, respectively. These peaks can readily correspond to NiFe_2_O_4_ (JCPDS No.10−0325), suggesting the high purity of the prepared NFO-YS and NFO-S [42]. The XRD spectrum of NFO-YS@C is almost the same as that of NFO-YS and NFO-S. No evident peaks corresponding to carbon are found, indicating that the phase composition of the three materials is similar, and the carbon coating covered is an amorphous structure [51]. Figure 5b shows the Raman spectra of NFO-YS, NFO-S, and NFO-YS@C in the range of 200–2500 cm^−1^. A resonance characteristic of NiFe_2_O_4_ was observed (400−800 cm^−1^) in the NFO-YS and NFO-S [40,52]. Compared with NFO-YS and NFO-S, the Raman spectrum of NFO-YS@C shows two evident peaks at 1350 and 1600 cm^−1^ after being coated with carbon. Among them, the peak at 1350 cm^−1^ is labeled as the D (disordered) band, whereas the peak at 1600 cm^−1^ is marked as the G (graphite) band. The R-value is the relative intensity ratio (ID/IG) of D peak to G peak, which represents the degree of graphitization of carbon [53,54]. Here, the R-value of NFO-YS@C was calculated to be 0.84, indicating that the carbon in the NFO-YS@C is amorphous carbon.

**Figure 6:**
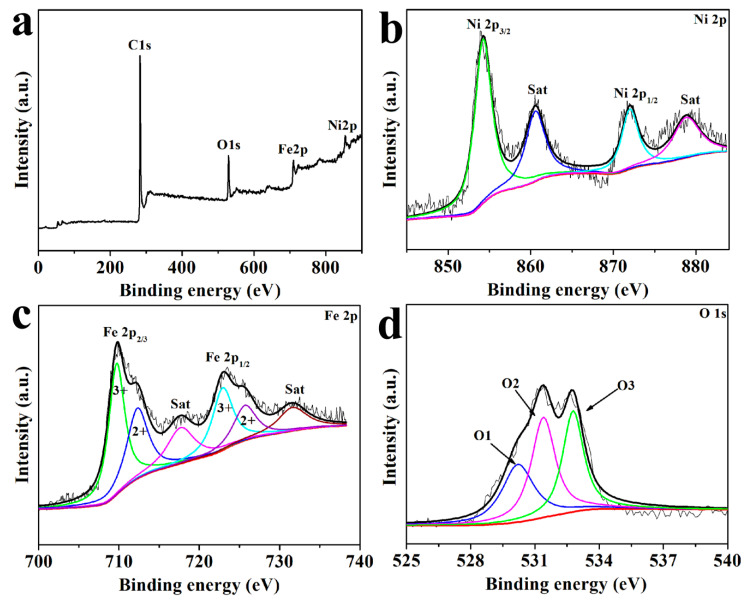
XPS spectra of (**a**) survey scan, (**b**) Ni, (**c**) Fe, and (**d**) O for NFO-YS@C.

The XPS survey spectrum in Figure 6a proves that Ni, Fe, O, and C are present in NFO-YS@C. No other peaks can be seen in the survey spectrum, indicating that the prepared NFO-YS@C has high purity. This result corresponds to the conclusion of the XRD and element mapping images. Four distinct peaks can be observed in the Ni 2p spectrum (Figure 6b). The peaks of Ni 2p3/2 and Ni 2p1/2 appear at 854.3 and 860.6 eV, respectively. The peaks at 878.9 and 872.8 eV are attributed to shakeup satellites [55]. In the Fe 2p spectrum (Figure 6c), Fe 2p2/3 has two spin-orbit peaks at 709.8 and 712.4 eV, which are attributed to Fe^3+^ and Fe^2+^, respectively. The peak of Fe 2p1/2 at 723.0 eV is attributed to Fe^3+^, whereas that at 725.8 eV is attributed to Fe^2+^. The shakeup satellite peaks of Fe 2p are at 717.8 and 731.8 eV. The results show that the metal valence of NFO-YS contains Ni^2+^, Fe^2+^, and Fe^3+^. The O 1s spectrum in Figure 6d reveals three peaks at 530.2, 531.4, and 532.8 eV, suggesting that the species in the NFO-YS have three types of oxygen. The O1 peak indicates the chemical bonding between oxygen and metal atoms, the O2 peak is attributable to oxygen vacancies, and the O3 peak is related to physisorbed and chemisorbed water on the surface [56]. The spectrum of C1s is shown in Figure S4. The peaks located at the 284.6 are originated from the C=C bond, while the peaks at 285.6, 286.3 and 288.4eV are attributed to the C−O, C=O and O−C=O bonds [57], which proves the existence of various oxygen-containing functional groups on the surface of NYO-YS@C. The presence of oxygen-containing functional groups can enhance the wettability of the surface and electrolyte and improve the electrochemical performance [49]. The TGA curve of NFO-YS@C in Figure S5 shows that the weight of NFO-YS@C has a large loss between 400 °C and 550 °C, and the weight retention rate decreases from 97.7% to 85.8%, indicating that the carbon content is about 11.9%.

**Figure 7:**
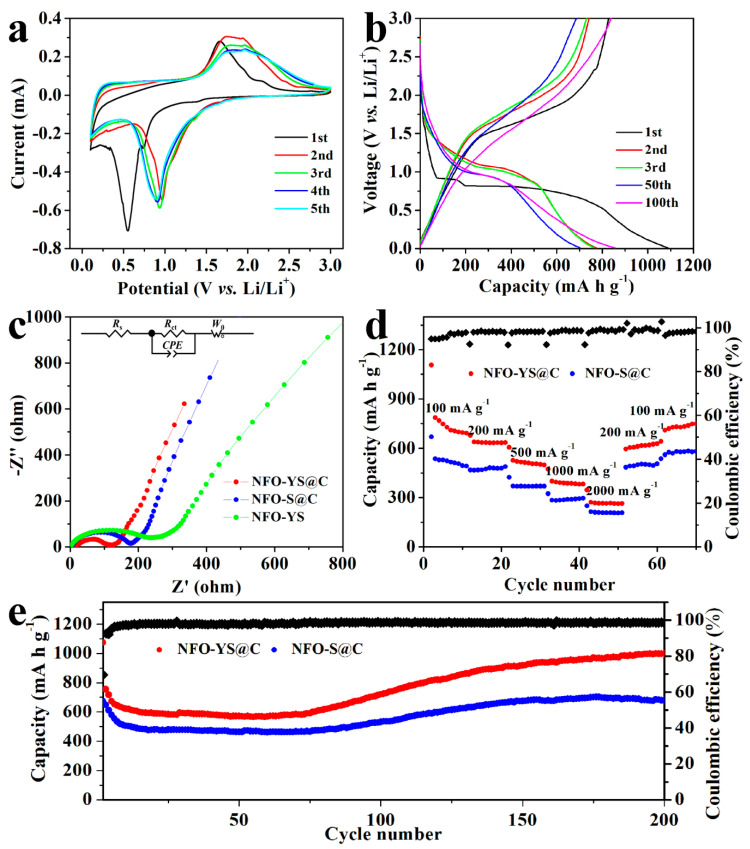
(**a**) CV curves of NFO-YS@C in the first five cycles; (**b**) the discharge-charge curves of NFO-YS@C at 100 mA g^−1^; (**c**) Nyquist plots of the NFO-YS, NFO-S@C, and NFO-YS@C; (**d**) rate capability of NFO-S@C and NFO-YS@C; (**e**) the cycling performances and Coulombic efficiencies at 200 mA g^−1^ of NFO-S @C and NFO-YS@C.

The lithium storage mechanism of the NFO-YS@C nanospheres as the anode was studied by using CV. The CV curve of the first five circles is shown in Figure 7a. In the first cathodic polarization, an obvious peak at 0.6 V corresponds to the reduction of NiFe_2_O_4_ to Ni and Fe with the simultaneous formation of Li_2_O. The corresponding chemical reaction mechanism of the first reduction curve is the following equation [58]:

8Li^+^ + NiFe_2_O_4_ + 8e^−^ → Ni + 2Fe + 4Li_2_O (1)

In the next cycle, the peak potential of the reduction peak was shifted from a low potential of 0.6 V to a high potential of near 1.0 V, and the peak intensity decreased substantially. This phenomenon is due to the capacity loss caused by the irreversible reaction in the 1st cycle [59]. The oxidation peak of the first cycle curve exists at about 1.7 V, corresponding to the oxidation of metals Fe and Ni [60]. The reaction equations can be expressed as follows:

Ni + Li_2_O ↔ NiO + 2Li^+^ + 2e^−^ (2)

2Fe + 3Li_2_O ↔ Fe_2_O_3_ + 6Li^+^ + 6e^−^ (3)

In the subsequent cycle, the oxidation peak at 1.7 V moved to 1.8 V due to the polarization of the battery [61]. Nearly overlapping characteristic peaks and similar CV curves were observed from subsequent cycles, implying that the NFO-YS@C nanospheres have superior reversibility and excellent cyclic stability [62]. Figure 7b shows the charge–discharge curves of the NFO-YS@C at a current density of 100 mA g^−1^. In the 1st cycle, the discharge plateau was located at 0.6 V, corresponding to the position of the cathode peak of the CV curve, which is caused by the reaction of the reduction reaction of Ni^2+^ and Fe^3+^ to Ni^0^ and Fe^0^. The platform was subsequently increased to about 1.0 V due to the generation of the SEI layer [52,60]. In addition, the initial discharge capacity of NFO-YS@C was 1087.1 mA h g^−1^, while the charge capacity is 828.4 mA h g^−1^, corresponding to a Coulombic efficiency (CE) of 76%. The 24% capacity loss is due to the irreversible reaction and decomposition of the electrolyte forming the SEI film [63,64]. Subsequently, the discharge capacity of the NFO-YS@C electrode gradually decayed during the early stage and dropped to 702.3 mA h g^−1^ at the 50th cycle. Interestingly, the specific capacity of 100 cycles increased to 855.9 mA h g^−1^. This phenomenon may be ascribed to the activation of the NFO-YS@C electrode at a low current [58].

EIS of NFO-YS, NFO-YS@C, and NFO-S@C electrodes are shown in Figure 7c. Arcs in the high-middle-frequency range correspond to the charge transfer resistance (*R*_ct_). Slope lines at a low frequency can be attributed to the Warburg resistance (*W*_0_) of Li-ion diffusion in electrode materials [61,65]. Figure 7c shows that among the three samples, the semicircle of NFO-S@C is the smallest, proving that the carbon coating and yolk-shell structure are conducive to the transfer of electron and Li-ion, which will result in excellent conductivity and low resistance, and improve electrochemical performance. The *R*_ct_ of NFO-S without carbon coating is significantly bigger than that of NFO-YS@C and NFO-S@C. This may be because the carbon coating improves the electronic conductivity of the material and forms a stable SEI film structure on the surface [66,67]. This indicates that the carbon coating has an important influence on the improvement of electrochemical performance. Next, the rate capabilities of NFO-YS@C and NFO-S@C anode materials at various current densities were evaluated. NFO-YS@C shows better rate capability in Figure 7d. When the current densities were 100, 200, 500, 1000, and 2000 mA g^−1^, NFO-YS@C can achieve high specific capacitances of 727.4, 638.3, 526.7, 400.4 and 271.2 mA h g^−1^, respectively. Rechecking the recovered specific capacity with a low current density, the capacity rapidly increased to 796.7 mA h g^−1^, which proves that NFO-YS@C electrode has good reversibility. By contrast, the NFO-S@C only had low capacities of 523.3, 466.9, 369.9, 282.2, and 206.0 mA h g^−1^. The results demonstrate that NFO-YS@C has higher capacities at different current densities than NFO-S@C and exhibits excellent rate capability, which implies that NFO-YS@C has great advantages in composition and structure.

Figure 7e shows the cycling performances of NFO-YS@C and NFO-S@C nanospheres at 200 mA g^−1^. The initial discharge capacity of NFO-YS@C was 1074.5 mA h g^−1^ with an initial CE of 69.63%, and the CE rose to about 98.3% in subsequent cycles, indicating excellent electrochemical reversibility. In the first 60 cycles, the discharge capacity of NFO-YS@C slowly decreased to 566 mA h g^−1^, and then gradually increased to 1023.5 mA h g^−1^. This phenomenon can be attributed to the reactivation caused by the degradation of the electrolyte resulting in the growth of the gel-like polymer layer [59,68]. A similar situation occurred in other transition metal oxides [69,70]. By contrast, NFO-S@C exhibited a capacity of only 676.6 mA h g^−1^ in the 200 cycles. The results prove that the yolk-shell sphere structure has more advantages than the solid sphere structure in terms of cycle stability, probably because the yolk-shell structure has short ion/electron transmission paths, multiple active sites, and excellent ability to adapt to volume strain. The structural advantages of yolk-shell structure and carbon coating allow NFO-YS@C nanospheres to have high reversible specific capacity and stable cycling performance. This result fully shows that the excellent complex hollow structure design has an important influence on the improvement of electrochemical performance.

This structure with the yolk-shell and carbon coating showed higher electrochemical performance in this work, and compared with some related works previously reported, it also has high reversible capacity and capacity retention rate. Table 1 lists the electrochemical performance including this work and the previously reported works. Compared with the materials in the table, the NFO-YS@C nanospheres prepared in this work show the best specific capacity for the following possible reasons. First, the yolk-shell structure of NiFe_2_O_4_ effectively shortens the Li^+^/electron diffusion pathways during the charging/discharging and increases the electrode/electrolyte contact area. Second, the yolk-shell structure can buffer the strain caused by Li^+^ deintercalation. Third, the carbon shell can effectively improve the conductivity of the material, and the rich mesoporous structure of NFO-YS@C can provide a high specific surface area, increase penetration of electrolyte, and accelerate the dynamic process of ion diffusion. NFO-YS@C nanospheres such as LIB anode materials exhibit high specific capacity, excellent rate performance, and good stability due to the unique NiFe_2_O_4_ yolk-shell and carbon shell structures.

## 4. Conclusions

In summary, we synthesized spinel NFO nanospheres with a controllable internal structure (including yolk-shell and solid structure) through a facile strategy. Then, a uniform carbon coating was formed after resorcinol and formaldehyde resin were carbonized. The electrochemical performance of the prepared NFO-YS@C and NFO-S@C as LiB anode materials showed that NFO-YS@C exhibits better electrochemical performance, including high initial capacity (1087.1 mA h g^−1^ at 100 mA g^−1^), excellent cycle stability (95.3% capacity retention after 200 cycles at 200 mA g^−1^), and superior rate capability. The outstanding electrochemical performance may benefit from the unique void structure of the yolk-shell and the high conductivity of the carbon coating. In addition, the current work may provide several ideas for the feasible synthesis of complex nanostructures and can be extended to synthesize other complex structures and components.

## Figures and Tables

**Table 1:** The comparison of electrochemical performances delivered by the anode in this work and those in previous studies.

Materials	Current Densies (mA g^−1^)	Cycle Numbers	Reversible Capacities (mA h g^−1^)	Capacity Retention (%)	Refs
NiFe_2_O_4_/C	100	50	892.4	69.8	[71]
NiFe_2_O_4_/Si	100	100	906	57.5	[72]
NiFe_2_O_4_	500	100	786	85.2	[42]
NiFe_2_O_4_/graphene	100	50	805	71.4	[23]
NiFe_2_O_4_/CNTs	100	100	624.6	46.3	[63]
NiFe_2_O_4_/expanded graphite	100	120	800	67.7	[61]
NiFe_2_O_4_/C	915	100	381.8	29.3	[73]
NiFe_2_O_4_/graphite	200	300	963.4	86.3	[40]
NFO-YS/C	200	200	1074.5	95.3	This work

## Supplementary Materials

The following are available online at https://www.mdpi.com/2079-4991/10/10/1994/s1, Figure S1: FESEM images of the NFO-S@C with (a) high- and (b) low- magnification, Figure S2: TEM images of the NiFe-glycerate precursors with (a) high- and (b) low- magnification, Figure S3: TEM image of the NFO-S@C, Figure S4: XPS spectra of C 1s for the sample of NFO-YS@C, Figure S5: TGA curve of NFO-YS@C.
