# Supplementary material for: Preparations of NiFe2O4 Yolk-Shell@C Nanospheres and Their Performances as Anode Materials for Lithium-Ion Batteries
Source: Nanomaterials (Basel). 2020 Oct 9;10(10):1994. doi: 10.3390/nano10101994 (PMC7600623; doi:10.3390/nano10101994)
Supplement: Supplementary file 1 [file nanomaterials-10-01994-s001.pdf]

nanomaterials

MDPI

Supplementary Materials

Preparations of $NiFe_2O_4$ Yolk-Shell@C Nanospheres and Their Performances as Anode Materials for Lithium-Ion Batteries

Tianli Liu, Qinghua Gong, Pei Cao, Xuefeng Sun, Jing Ren, Shaonan Gu * and Guowei Zhou *

Key Laboratory of Fine Chemicals in Universities of Shandong, School of Chemistry and Chemical Engineering, Qilu University of Technology (Shandong Academy of Sciences), Jinan 250353, China; 17862979520@163.com (T.L.); 18396814931@163.com (Q.G.); caopei8956@126.com (P.C.); sunxf0210@163.com (X.S.); 17862963042@163.com (J.R.)

* Correspondence: sngu@qlu.edu.cn (S.G.); gwzhou@qlu.edu.cn or guoweizhou@hotmail.com (G.Z.); Tel.: +86-531-89631696 (G.Z.)

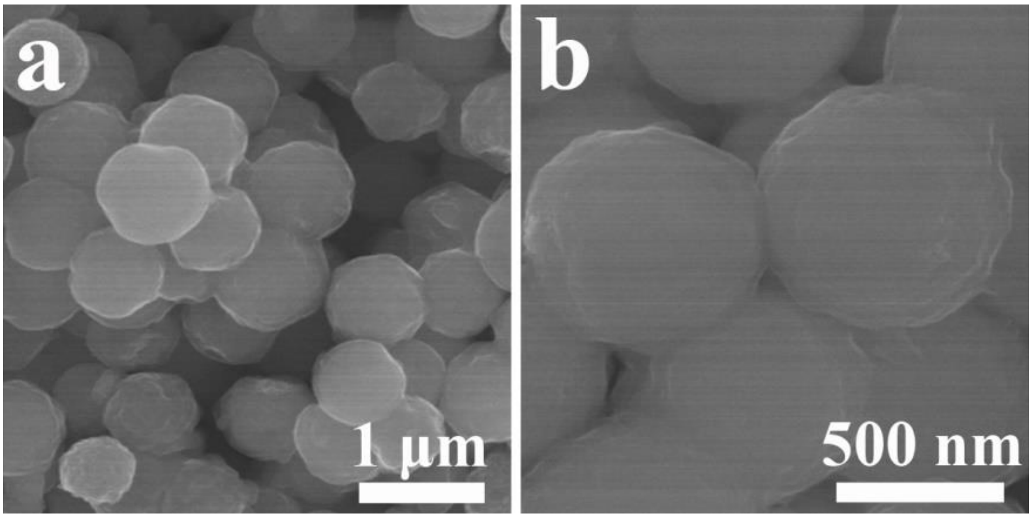

Figure S1. FESEM images of the NFO-S@C with (**a**) high- and (**b**) low- magnification.

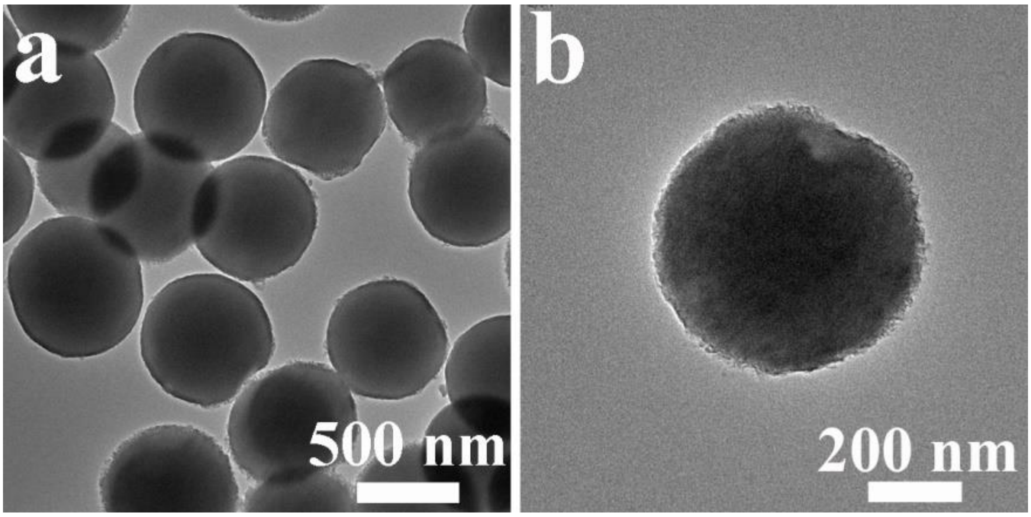

Figure S2. TEM images of the NiFe-glycerate precursors with (**a**) high- and (**b**) low- magnification.

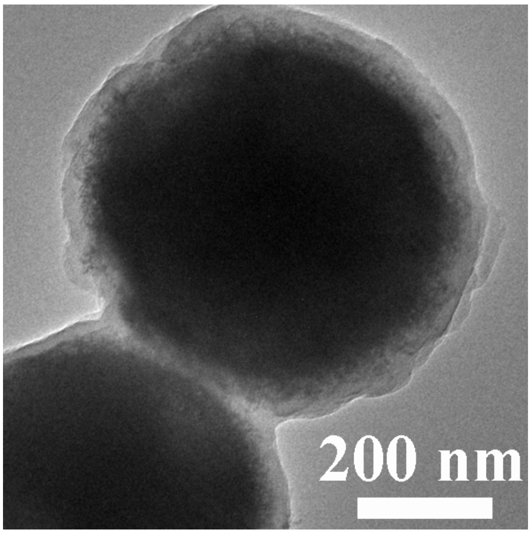

Figure S3. TEM image of the NFO-S@C.

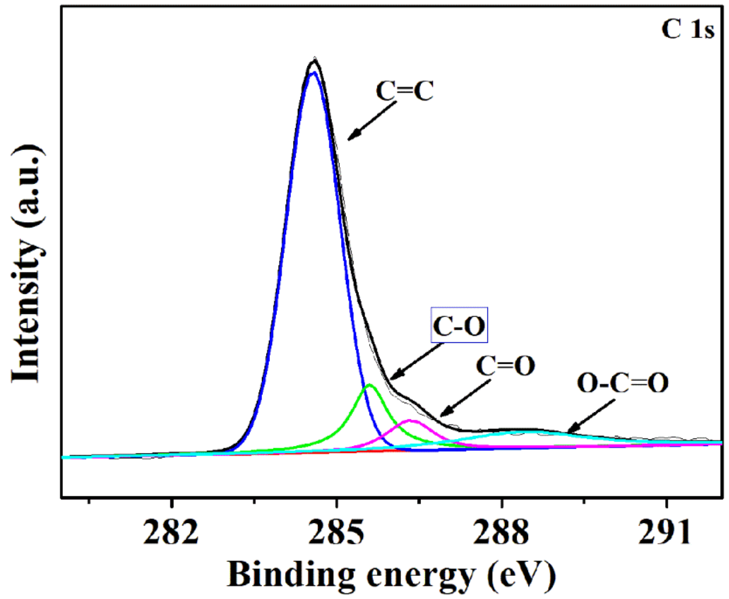

Figure S4. XPS spectra of C 1s for the sample of NFO-YS@C.

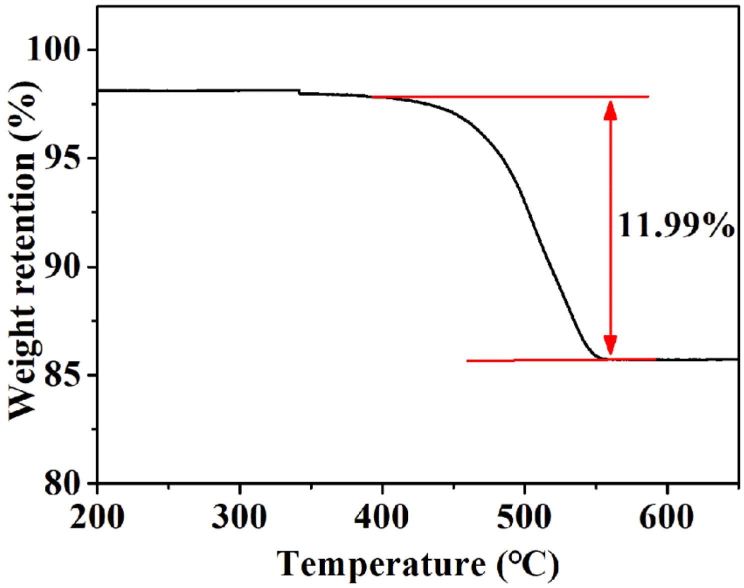

Figure S5. TGA curve of NFO-YS@C.
